# A 2-Min Transient Ischemia Confers Cerebral Ischemic Tolerance in Non-Obese Gerbils, but Results in Neuronal Death in Obese Gerbils by Increasing Abnormal mTOR Activation-Mediated Oxidative Stress and Neuroinflammation

**DOI:** 10.3390/cells8101126

**Published:** 2019-09-22

**Authors:** Joon Ha Park, Ji Hyeon Ahn, Minah Song, Hyunjung Kim, Cheol Woo Park, Young Eun Park, Tae-Kyeong Lee, Jae-Chul Lee, Dae Won Kim, Choong-Hyun Lee, In Koo Hwang, Bing Chun Yan, Sungwoo Ryoo, Young-Myeong Kim, Il Jun Kang, Moo-Ho Won, Soo Young Choi

**Affiliations:** 1 Department of Anatomy, College of Korean Medicine, Dongguk University, Gyeongju, Gyeongbuk 38066, Korea; parkfamilyda@hanmail.net; 2 Department of Biomedical Science and Research Institute for Bioscience and Biotechnology, Hallym University, Chuncheon, Gangwon 24252, Korea; jh-ahn@hallym.ac.kr; 3 Center for Virus Research and Testing, Korea Research Institute of Chemical Technology, Daejeon 34114, Korea; zlscydn@naver.com; 4 Knotus Co. Ltd., Incheon 22014, Korea; nicolehkim@naver.com; 5 Department of Neurobiology, School of Medicine, Kangwon National University, Chuncheon, Gangwon 24341, Korea; flfhflfh@naver.com (C.W.P.); taeparo@naver.com (Y.E.P.); xorud312@naver.com (T.-K.L.); anajclee@kangwon.ac.kr (J.-C.L.); 6 Department of Biochemistry and Molecular Biology, and Research Institute of Oral Sciences, College of Dentistry, Gangneung-Wonju National University, Gangneung, Gangwon 25457, Korea; kimdw@gwnu.ac.kr; 7 Department of Pharmacy, College of Pharmacy, Dankook University, Cheonan, Chungnam 31116, Korea; anaphy@dankook.ac.kr; 8 Department of Anatomy and Cell Biology, College of Veterinary Medicine and Research Institute for Veterinary Science, Seoul National University, Seoul 08826, Korea; vetmed2@snu.ac.kr; 9 Jiangsu Key Laboratory of Integrated Traditional Chinese and Western Medicine for Prevention and Treatment of Senile Diseases, Medical college of Yangzhou University, Yangzhou 225001, China; bcyan@yzu.edu.cn; 10 Department of Biological Sciences, College of Natural Sciences, Kangwon National University, Chuncheon, Gangwon 24341, Korea; ryoosw08@kangwon.ac.kr; 11 Department of Molecular and Cellular Biochemistry, School of Medicine, Kangwon National University, Chuncheon, Gangwon 24341, Korea; ymkim@kangwon.ac.kr; 12 Department of Food Science and Nutrition, Hallym University, Chuncheon, Gangwon 24252, Korea; ijkang@hallym.ac.kr

**Keywords:** obesity, brief transient ischemia, neuronal death, oxidative stress, neuroinflammation, mammalian target of rapamycin

## Abstract

A brief episode of transient ischemia (TI) can confer cerebral ischemic tolerance against a subsequent severer TI under standard condition. The brain under obesity’s conditions is more sensitive to ischemic injury. However, the impact of a brief episode of TI under obesity’s conditions has not been fully addressed yet. Thus, the objective of this study was to determine the effect of a brief TI in the hippocampus of high-fat diet (HFD)-induced obese gerbils and related mechanisms. Gerbils were maintained on HFD or normal diet (ND) for 12 weeks and subjected to 2 min TI. HFD gerbils were heavier, with higher blood glucose, serum total cholesterol, triglycerides, and leptin levels. Massive loss of pyramidal neurons occurred in the hippocampal cornu ammonis 1 (CA1) field of HFD animals at 5 days after 2 min of TI, but 2 min of TI did not elicit death of pyramidal neurons in ND gerbils. The HFD group showed significantly increased levels of oxidative stress indicators (dihydroethidium and 4-hydroxynonenal) and proinflammatory cytokines (tumor necrosis factor-α and interleukin-1β) and microglial activation in pre- and/or post-ischemic phases compared to the ND group. Levels of mammalian target of rapamycin (mTOR) and phosphorylated-mTOR in the CA1 field of the HFD group were also significantly higher than the ND group. On the other hand, inhibition of mTOR activation by rapamycin (an allosteric mTOR inhibitor) significantly attenuated neuronal death induced by HFD, showing reduction of HFD-induced increases of oxidative stress indicators and proinflammatory cytokines, and microglia activation. Taken together, a brief episode of TI can evoke neuronal death under obesity’s conditions. It might be closely associated with an abnormal increase of mTOR activation-mediated, severe oxidative stress and neuroinflammation in pre- and/or post-ischemic phases.

## 1. Introduction

Transient ischemia (TI) in the whole brain, following a complete interruption of blood flow can lead to irreversible neuronal damage/death in vulnerable brain areas [1,2]. The CA1 field (CA1) in the hippocampus is known to be especially sensitive to TI [1]. Extensive loss of pyramidal neurons in the CA1 occurs typically over several days after TI. It can result in hippocampal-dependent cognitive deficits [3,4]. Although many studies have suggested that oxidative stress and neuroinflammation are crucial factors involved in ischemia-induced neuronal death [5,6,7], underlying mechanisms related to this phenomenon have not been clearly established yet.

Clinically, a brief TI in brains can trigger some neuroprotection against subsequent severe episode of TI by inducing ischemic tolerance [8,9]. In that regard, researchers have demonstrated that a brief duration (1–3 min) of TI can prevent neuronal death in CA1 against a subsequent, longer TI that can result in massive loss of CA1 pyramidal neurons in rats [10,11], mice [12], and gerbils [13,14].

Many factors affect the pathophysiology in ischemic brains [15]. Among these factors, high fat diet (HFD)-induced obesity is a significant and serious public health problem. It is a global epidemic according to the World Health Organization [16]. Obesity is known to increase the morbidity and mortality following a variety of diseases, including cardio-cerebrovascular disease. It is considered a major factor that can lead to worse outcomes after ischemic insults [17]. Recently, preclinical studies have shown that prolonged diet-induced obesity can exacerbate TI-induced brain injury in rats [18,19], mice [20,21], and gerbils [22]. However, the underlying mechanisms of obesity-enhanced ischemic brain injury remain unclear.

Mammalian target of rapamycin (mTOR) is a highly conversed serine/threonine protein kinase that regulates important cellular functions, including cell growth, metabolism, proliferation, and survival/death [23]. It is involved in a variety of pathophysiological processes, such as oxidative stress and inflammation, in response to stress stimuli [24,25]. Thus, the role of the mTOR signaling pathway in pathophysiological processes of critical illness has received increasing attention by many researchers. Accumulating evidence has shown that an abnormal mTOR signaling pathway plays a role in metabolic and neurological disorders, including obesity and brain ischemia [26,27].

Although some studies have shown that brains of HFD-induced obese animals are more sensitive to ischemic injury, the impact of a brief episode of TI, known to induce cerebral ischemic tolerance in normal animals, has not yet been reported in animal models of HFD-induced obesity. Therefore, the objective of this study was to investigate effects of a brief episode (2 min) of TI in the hippocampus of HFD-induced obese gerbils, and related mechanisms. The HFD-induced obese gerbil was chosen for this study because it is a well-established animal model for studying TI-induced neuronal damage/death and its related mechanisms [6,28].

## 2. Materials and Methods

### 2.1. Experimental Animals and Diets

Male Mongolian gerbils *(Meriones unguiculatus*) were used at 6 months (body weight, 70–80 g) of age. Experimental procedure of this study was approved (approval number, KW-180124-1) by the Institutional Animal Care and Use Committee at Kangwon University (Chuncheon, Korea). Animals were fed a commercially available rodent diet, which consisted of different fat concentrations as follows: normal diet (ND, D12450B, 10 kcal% fat, 20 kcal% protein, 70 kcal% carbohydrate, Research Diets, NJ, USA) and HFD (D12492, 60 kcal% fat, 20 kcal% protein, 20 kcal% carbohydrate, Research Diets). Animals were allowed free access to food and water for 12 weeks. Body weight was measured weekly.

To study effects of 2 min of TI and its related mechanisms in ND-fed and HFD-fed animals, gerbils, were divided into 6 groups: (1) ND sham group (*n* = 14), which was given no ischemia in ND-fed gerbils; (2) ND 2-min TI group (*n* = 28), which was given 2 min of TI in ND-fed gerbils and sacrificed at 2 (*n* = 14) and 5 days (*n* = 14) after TI; (3) HFD sham group (*n* = 14), which was given no ischemia in HFD-fed gerbils; (4) HFD 2-min TI group (*n* = 28), which was given 2 min of TI in HFD-fed gerbils and sacrificed at 2 (*n* = 14) and 5 days (*n* = 14) after TI; (5) HFD/RAPA sham group (*n* = 14), which was given rapamycin (RAPA) and no ischemia in HFD-fed gerbils; and (6) HFD/RAPA 2-min TI group (*n* = 28), which was given RAPA and 2 min of TI in HFD-fed gerbils and sacrificed at 2 (*n* = 14) and 5 days (*n* = 14) after TI.

### 2.2. Treatment with RAPA

**Figure 1:**
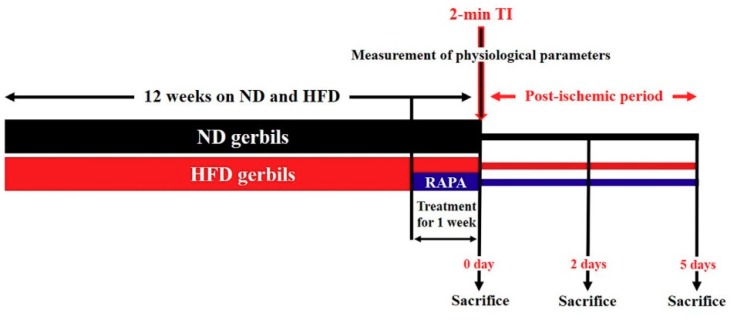
Experimental design. Normal diet (ND) or high-fat diet (HFD) was administered for 12 weeks, and RAPA was administered to HFD-fed gerbils for the last 1 week. At 12 weeks after the feeding, physiological parameters (body weight, epididymal fat weight, blood glucose levels, serum lipid concentrations, and leptin levels) were measured. After the measurement, the gerbils were subjected to a 2-min TI and sacrificed at sham, 2 days, and 5 days after the ischemic surgery for various analyses.

We evaluated whether inhibition of abnormal mTOR activation by administration of RAPA (a highly selective mTOR inhibitor) attenuated neuronal death/damage in HFD-induced obese gerbils following 2 min of TI, by inhibiting severe oxidative stress and neuroinflammation. RAPA (6 mg/kg, Sigma-Aldrich, St. Louis, MO, USA), an allosteric mammalian target of rapamycin (mTOR) inhibitor, was dissolved in dimethyl sulfoxide, and the final concentration (less than 2%) was adjusted using normal saline. RAPA was administered intraperitoneally once a day for the last 1 week during HFD treatment in the HFD/RAPA groups, as shown in Figure 1. Animals in the HFD groups were administered the same concentration of solvent, dimethyl sulfoxide, and saline, as a vehicle control. The dose and injection duration of RAPA was selected based on a previous study that showed that effects of RAPA against ischemic brain injury in acute hyperglycemic rats [29].

### 2.3. Analyses of Glucose Levels, and Lipid and Leptin Profiles

As we described previously [30], at 12 weeks after feeding ND, HFD, or HFD/RAPA, the animals (*n* = 14/group) were anesthetized with 60 mg/kg Zoletil 50^®^ (Virbac, Carros, France, i.p.). A blood sample was collected from each animal by orbital puncture, and the blood glucose level was analyzed by using a blood glucose monitor (Ascensia Elite XL Blood Glucose Meter, Bayer, Toronto, ON, Canada). Serum was separated from the blood by centrifugation at 12,000 *g* for 20 min at 4 °C (centrifuge 5424R; Eppendorf, Hamburg, Germany), and the serum was stored at –80 °C until analysis. Total cholesterol and the triglyceride level in serum was measured enzymatically using a dry chemistry analyzer (FUJI DRI-CHEM NX500; Fujifilm, Tokyo, Japan). In addition, serum leptin level was determined by radioimmunoassay with a multi-species kit (XL-85K; Linco Research, St Charles, MO, USA). The lowest level of leptin to be able to be detected by this assay was 1.0 ng/mL, when we used a 100-mL sample size (instructions for multi-species leptin radioimmunoassay kit). Finally, the epididymal fat depot was carefully removed, rinsed with saline, and then weighed.

### 2.4. Induction of 2-min TI

TI was induced according to our published method [31]. In brief, animals were anesthetized with a mixture of 2.5% isoflurane in 33% oxygen, and 67% nitrous oxide. Both common carotid arteries were occluded for 2-min TI. The restoration of blood flow (reperfusion) was directly observed under an ophthalmoscope (HEINE K180, Heine Optotechnik, Herrsching, Germany). Body (rectal) temperature was maintained at normothermic (37 ± 0.5 °C) condition during the surgery. Sham animals received the same surgical procedure except the ligation of common carotid arteries.

### 2.5. Tissue Section for Histology

As we described previously [31], animals (*n* = 7 at sham, 2 days and 5 days after ischemic surgery) were anesthetized with 70 mg/kg of pentobarbital sodium (JW Pharm. Co., Ltd., Daegu, Korea, i.p.) and perfused transcardially with 4% paraformaldehyde (in 0.1 M phosphate-buffer, pH 7.4). Their brains were removed and more fixed in that same fixative for 6 h. Tissues containing the hippocampus were serially sectioned into 30-μm coronal sections in a cryostat (Leica, Wetzlar, Germany).

### 2.6. Cresyl Violet Staining

To examine effects of 2 min of TI in the hippocampus of ND-fed and HFD-fed gerbils, the sections were stained with cresyl violet (CV) as we descried previously [31]. In brief, we made 1.0% (*w*/*v*) CV acetate (Sigma-Aldrich), and added glacial acetic acid (Sigma-Aldrich). The sections were stained with the solution for 2 min at room temperature and washed twice in distilled water. The stained sections were dehydrated by immersing in 50%, 70%, 80%, 90%, 95%, and 100% ethanol baths in succession, at room temperature. After dehydration, the sections were mounted with Canada balsam (Kanto, Tokyo, Japan).

### 2.7. NeuN Immunofluorescence and F-J B Histofluorescence Staining

To investigate neuronal damage/death in the CA1 of ND-fed and HFD-fed gerbils after 2 min of TI, neuron-specific soluble nuclear antigen (NeuN, a marker for neurons) immunofluorescence and Fluoro-Jade B (F-J B, a high affinity fluorescent marker for neuronal degeneration) histofluorescence staining were done according to previously published procedure [6]. In brief, the sections were incubated with mouse anti-NeuN (1:1000, Chemicon, Temecula, CA, USA) overnight at room temperature and reacted with Cy3-conjugated donkey anti-mouse immunoglobulin G (IgG, 1:500, Vector Laboratories Inc., Burlingame, CA, USA) for 2 h at room temperature. Their immunoreactions were observed under an epifluorescence microscope (BX53; Olympus Deutschland GmbH, Hamburg, Germany). For F-J B staining, the sections were immersed in a solution of 1% sodium hydroxide (Sigma-Aldrich), transferred to a solution of 0.06% potassium permanganate (Sigma-Aldrich, St. Louis, MO, USA), and, finally, reacted with 0004% F-J B (Histochem, Jefferson, AR, USA) solution. The stained sections were washed and placed on a slide warmer (approximately 50 °C). We examined the sections under an epifluorescence microscope (BX53; Olympus) with blue excitation light (450–490 nm).

### 2.8. Immunohistochemistry

Immunohistochemistry was carried out for (1) lipid peroxidation using 4-hydroxy-2-nonenal (HNE); (2) microglial activation using ionized calcium binding adapter molecule 1 (Iba-1); (3) proinflammatory responses, using interleukin (IL)-1β and tumor necrosis factor (TNF)-α; and (4) mTOR activation, using mTOR and phosphorylated mTOR (p-mTOR) as primary antibodies.

The sections at sham, 2 days, and 5 days after the ischemic surgery were immunohistochemically stained to our published procedure [6,30]. In brief, the sections were incubated with each diluted antibody as follows: mouse anti-HNE (1:1,000, Alexis Biochemicals, San Diego, CA, USA), rabbit anti-Iba-1 (1:800, Wako, Osaka, Japan), rabbit anti-IL-1β (1:200, Santa Cruz Biotechnology, Santa Cruz, CA, USA), rabbit anti-TNF-α (1:1,000, Abcam, Cambridge, MA, USA), rabbit anti-mTOR (1:100; Santa Cruz Biotechnology), or rabbit anti-p-mTOR (1:100; Santa Cruz Biotechnology, Santa Cruz, CA, USA). The reacted sections were exposed to biotinylated goat anti-mouse or rabbit IgG (1:200, Vector Laboratories Inc., Burlingame, CA, USA) and streptavidin peroxidase complex (1:200, Vector, Burlingame, CA, USA). Finally, the reacted sections were visualized by staining with 3,3′-diaminobenzidine (Sigma-Aldrich, St. Louis, MO, USA). In order to establish the specificity of the immunostaining, each negative control for IL-1 β, TNF-α, mATOR, and p-mTOR resulted in the absence of immunoreactivity in all structures (Figure S1).

### 2.9. Dihydroethidium Fluorescence Staining

To evaluate the in situ production of superoxide anion, oxidative fluorescent dye dihydroethidium (DHE; Sigma-Aldrich, St. Louis, MO, USA) was used. The detection of superoxide anion radical was performed as described previously [6]. In brief, the sections were equilibrated in Krebs-HEPES buffer (130 mM NaCl, 5.6 mM KCl, 2 mM CaCl2, 0.24 mM MgCl2, 8.3 mM HEPES, and 11 mM glucose, pH 7.4) for 30 min at 37 °C. Fresh buffer containing DHE (10 μmol/L) was applied to the sections. The sections were coverslipped and incubated in a light-protected humidified chamber for 2 h at 37 °C. Finally, the sections were examined under an epifluorescence microscope (BX53; Olympus) with an excitation wavelength of 520–540 nm. 

### 2.10. Western Blot Analysis

To examine changes of proinflammatory response and mTOR activation in the hippocampal CA1 following 2 min of TI, animals (*n* = 7 at sham, 2 days and 5 days after ischemic surgery) were used for western blot analysis according to the method described in our previous study [3,30]. In brief, the gerbils were anesthetized with 70 mg/kg pentobarbital sodium (JW Pharm, Seoul, Republic of Korea), and their brains were serially and transversely cut into 400 μm thick sections on a vibratome (VP1000P; Leica), and CA1 was dissected with a surgical blade. Tissues of CA1 were homogenized in 50 mM PBS (pH 7.4) containing ethylene glycol tetraacetic acid (pH 8.0), 0.2% Nonidet P-40, 10 mM ethylenediaminetetraacetic acid (pH 8.0), 15 mM sodium pyrophosphate, 100 mM β-glycerophosphate, 50 mM sodium fluoride, 150 mM sodium chloride, 2 mM sodium orthvanadate, 1 mM phenylmethylsulfonyl fluoride, and 1 mM dithiothreitol (DTT). The homogenized tissues were centrifuged at 15,000 × *g* for 25 min at 4 °C. Protein levels in the supernatants were determined using a micro bicinchoninic acid protein assay kit with bovine serum albumin as a standard (Pierce Chemical, Rockford, IL., USA). The aliquots containing 50 µg total protein were boiled in loading buffer containing 250 mM Tris (pH 6.8), 10 mM DTT, 10% sodium dodecyl sulfate, 0.5% bromophenol blue, and 50% Glycerol, and they were subsequently loaded onto a 10% polyacrylamide gel (Sigma-Aldrich). After the electrophoresis, the gels were transferred onto nitrocellulose membranes (Pall Corp., Pittsburgh, PA, USA). The membranes were subsequently incubated with diluted rabbit anti-IL-1β (1:200, Santa Cruz Biotechnology), rabbit anti-TNF-α (1:1,000, Abcam, Cambridge, MA, USA), rabbit anti-mTOR (1:200; Santa Cruz Biotechnology, Heidelberg, Germany), rabbit anti-p-mTOR (1:200; Santa Cruz Biotechnology, Heidelberg, Germany), and rabbit anti-β-actin (1:5,000, Sigma-Aldrich) overnight at 4 °C. Finally, they were exposed to peroxidase conjugated goat anti-rabbit IgG (1:4,000, Santa Cruz Biotechnology, Heidelberg, Germany) and an enhanced chemiluminescence kit (GE Healthcare Life Sciences, Chalfont, UK).

### 2.11. Data Analyses

To quantitatively analyze of neuronal death, three sections were selected with a 120 μm interval in each CA1 (anteroposterior –1.4 to –2.2 mm of the gerbil brain atlas) [32]. NeuN-positive (NeuN^+^) and F-J B^+^ cells were counted as previously described [31]. In short, digital images of the cells were obtained under an epifluorescence microscope (BX53; Olympus) equipped with digital camera (DP72; Olympus) connected to PC monitor. The cells were counted in a 250 × 250 μm square at the center of the CA1 under X20 primary magnification. Cell counts were obtained by averaging total numbers using an image analyzing system (software: Optimas 6.5, CyberMetrics, Scottsdale, AZ). 

To quantitatively analyze the density of HNE^+^, Iba-1^+^, IL-1β^+^, TNF-α^+^, mTOR^+^, and p-mTOR^+^ structures, we carried out according to our previous method [3]. In brief, digital images of the immunoreactive structures in the CA1 were taken like the above-mentioned method. The images were calibrated into an array of 512 × 512 pixels to a tissue area of 140 × 140 μm (40 × primary magnification). Density or immunoreactivity of the structures was evaluated on the basis of an optical density or immunoreactivity (OD or OI), which was obtained after transformation of the mean gray level using the formula: OD = log (256/mean gray level). The background was taken from areas adjacent to the measured area. Finally, after the background was subtracted, we compared them as a ratio of the relative optical density (ROD) for the Iba-1^+^ structure in the CA1 and/or relative immunoreactivity (RI) for HNE, IL-1β, TNF-α, mTOR, and p-mTOR in the CA1 pyramidal cells, which were calibrated as percentages using Adobe Photoshop version 8.0. Finally, they were analyzed using NIH Image 1.59 software. A ratio of the ROD or RI was calibrated as a percentage, with the ND sham group designated as 100%.

To measure the fluorescence intensity of DHE, a digital image was captured under an epifluorescence microscope (BX53; Olympus). The DHE fluorescence intensity was analyzed using Image-pro Plus 6.0 software. A ratio of the DHE fluorescence intensity was calibrated as a percentage, with the ND sham group designated as 100%.

Finally, we analyzed results of the western blotting according to our published procedure [6]. In brief, bands of IL-1β, TNF-α, mTOR, and p-mTOR obtained from the CA1 were scanned using ChemiDoc Imaging System (Bio-Rad Laboratories, Inc., Hercules, CA, USA). Densitometric analyses for the quantification of the bands were done using Scion Image software (Scion Corp., Frederick, MD, USA). Expression rates of the target proteins were normalized through corresponding expression rates of β-actin.

### 2.12. Statistical Analysis

Statistical power analysis for sample size estimation was performed using G* power 3.1 software (Dusseldorf, Germany). The sample size was at least seven gerbils per group, with an alpha error of 0.05 and a power of >80%. All statistical analyses were performed using GraphPad Prism (version 5.0; GraphPad Software, La Jolla, CA). Differences of the means among the groups were statistically analyzed by two-way analysis of variance (ANOVA) tests with post hoc Bonferroni's multiple comparison tests in order to elucidate ischemia-related differences among experimental groups. Statistical significance was considered at *p* < 0.05. Data are presented as the means ± standard errors of the mean (SEM).

## 3. Results

### 3.1. Changes in Physiological Parameters

**Figure 2:**
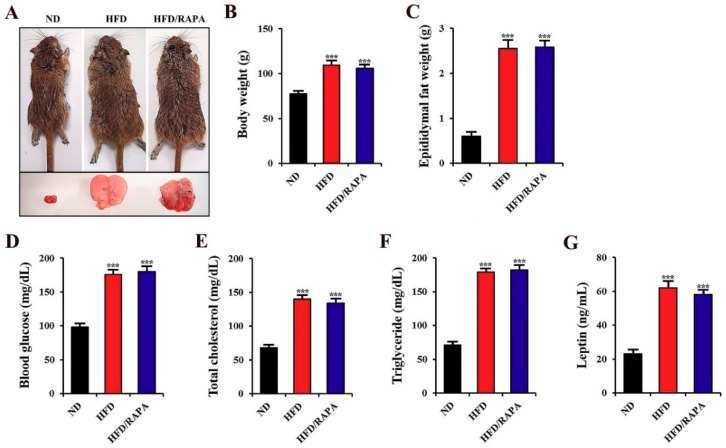
(**A**) Representative gerbils from the ND-fed group (left), HFD-fed group (middle), and HFD/RAPA-fed group (right), depicting gross images of whole body (top) and epididymal fat (bottom). (**B**–**G**) Physiological parameters, including body weight (**B**), epididymal fat weight (**C**), blood glucose (**D**), serum total cholesterol (**E**), triglyceride (**F**), and leptin levels (**G**). Note that no significant differences were shown between the HFD-fed and HFD/RAPA-fed groups. The bars indicate the means ± SEMs. *n* = 14/group. *** *p* < 0.001 versus ND-fed group.

Figure 2A shows representative gerbils fed either a ND, HFD, or HFD/RAPA, and their epididymal fat. In the HFD-fed gerbils, body weight, epididymal fat weight, blood glucose, serum total cholesterol, triglyceride, and leptin levels were significantly higher (*p* < 0.001) than those in the ND-fed group (Figure 2B–G). In the HFD/RAPA-fed gerbils, we did not find any significant changes in physiological parameters compared to those in the HFD-fed gerbils (Figure 2B–G).

### 3.2. Neuronal Death by 2 Min of TI in HFD-Fed, Obese Gerbils

#### CV^+^ Cells

**Figure 3:**
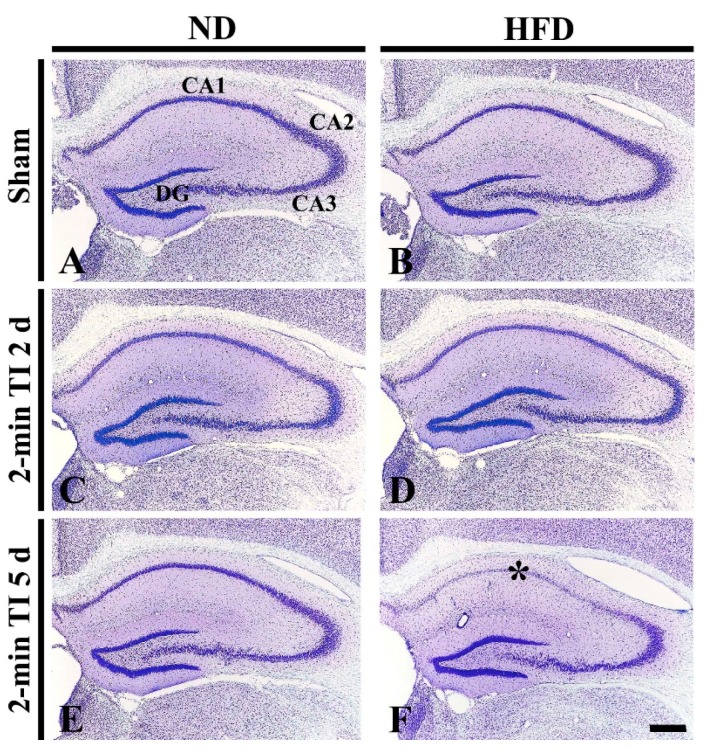
Cresyl violet (CV) staining in the hippocampus of the ND-fed group (left column) and HFD-fed group (right column) at sham (**A**,**B**), 2 days (**C**,**D**) and 5 days (**E**,**F**) after 2-min TI. In the HFD 2-min TI group, CV^+^ cells are very pale in the stratum pyramidale (f, asterisk) of the CA1 at 5 days post-ischemia. CA, cornus ammonis; DG, dentate gyrus. Scale bar = 400 μm.

CV staining was done to examine cellular changes in the hippocampus of the ND-fed non-obese and HFD-fed obese gerbils after 2 min of TI (Figure 3). In the ND sham group, intact CV^+^ cells were observed in all hippocampal subfields (Figure 3A). In the ND 2-min TI group, the distribution pattern of CV^+^ cells was not altered until 5 days post-ischemia (Figure 3C,E). In the HFD sham group, the distribution of CV^+^ cells in the hippocampus was similar to that in the ND sham group (Figure 3B). In the HFD 2-min TI group, although no significant change in CV^+^ cells was found at 2 days post-ischemia (Figure 3D), at 5 days post-ischemia, CV^+^ cells were very pale in the stratum pyramidale of the CA1 (Figure 3E): this means neuronal damage by 2 min of TI.

### 3.3. Neuroprotection by RAPA in HFD-Fed, Obese Gerbils 

#### NeuN^+^ and F-J B^+^ Cells

**Figure 4:**
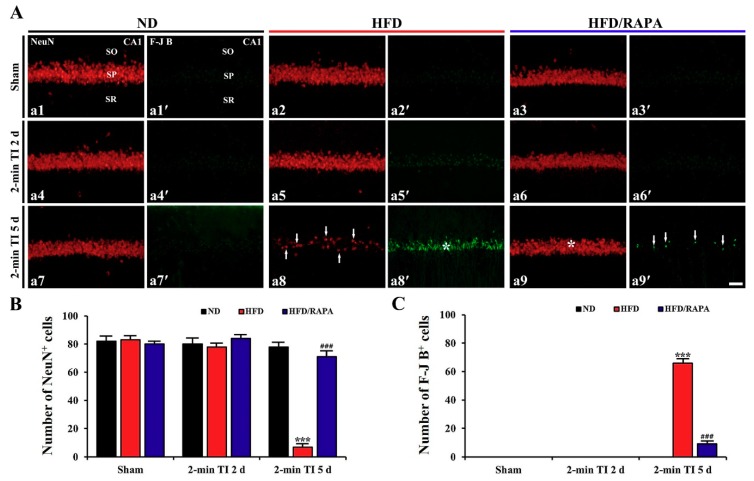
**(A**) NeuN immunofluorescence (a1–a9) and F-J B histofluorescence staining (a1′–a9′) in the CA1s of the ND-fed group (left column), HFD-fed group (middle column), and HFD/RAPA-fed group (right column) at sham (a1–a3, a1′–a3′), 2 days (a4–a6, a4′–a6′), and 5 days (a7–a9, a7′–a9′) after 2-min TI. In the HFD 2-min TI group, a few NeuN^+^ CA1 pyramidal cells (a8, arrows) and many F-J B^+^ CA1 pyramidal cells (a8′, asterisk) were observed at 5 days post-ischemia. However, at this time, abundant NeuN^+^ CA1 pyramidal cells (a9, asterisk) and a few F-J B^+^ CA1 pyramidal cells (a9′, arrows) were observed in the HFD/RAPA 2-min TI group. SO, stratum oriens; SP, stratum pyramidale; SR, stratum radiatum. Scale bar = 35 μm. (**B**,**C**) The mean numbers of NeuN^+^ (**B**) and F-J B^+^ (**C**) CA1 pyramidal cells (*n* = 7/group). The bars indicate the means ± SEMs. *** *p* < 0.001 versus each sham group; ^###^ *p* < 0.001 versus HFD 2-min TI group.

NeuN and F-J B were used for neuronal survival and degeneration, respectively, in the ND-fed non-obese and HFD-fed obese animals after 2 min of TI (Figure 4). In the ND sham group, pyramidal cells in the stratum pyramidale of the CA1, which are called CA1 pyramidal cells, were well immunostained with NeuN, and the cells were 82/250 × 250 μm (Figure 4A-a1,B). In this group, no F-J B^+^ CA1 pyramidal cells were detected (Figure 4A-a1′,C). In the ND 2-min TI group, the distribution pattern and numbers of NeuN^+^ and F-J B^+^ CA1 pyramidal cells were not different from those in the ND sham group (Figure 4A-a4-a4′-a7,4A-a7′,B,C). 

In the HFD sham group, the distribution pattern and numbers of NeuN^+^ and F-J B^+^ CA1 pyramidal cells were similar to those in the ND sham group (Figure 4A-a2,A-a2′,B,C). In the HFD 2-min TI group, no significant change in NeuN^+^ and F-J B^+^ CA1 pyramidal cells was observed at 2 days post-ischemia (Figure 4A-a5,4A-a5′,B,C): however, at 5 days post-ischemia, a significant decrease of NeuN^+^ CA1 pyramidal cells (7 cells/250 × 250 μm, *p* < 0.001) and a significant increase of F-J B^+^ CA1 pyramidal cells (66 cells/250 × 250 μm, *p* < 0.001) were both observed compared to those in the ND 2-min TI group (Figure 4A-a8,4A-a8′,B,C).

The distribution pattern and numbers of NeuN^+^ and F-J B^+^ CA1 pyramidal cells in the HFD/RAPA sham group were not significantly different from the ND sham and HFD sham groups (Figure 4A-a3,4A-a3′,B,C). In the HFD/RAPA 2-min TI group, no changes in NeuN^+^ or F-J B^+^ CA1 pyramidal cells were found at 2 days post-ischemia (Figure 4A-a6,4A-a6′,B,C), and, at 5 days post-ischemia, NeuN^+^ CA1 pyramidal cells were significantly increased (71 cells/250 × 250 μm, *p* < 0.001) and F-J B^+^ CA1 pyramidal cells were significantly decreased (9 cells/250 × 250 μm, *p* < 0.001) compared to those in the HFD 2-min TI group (Figure 4A-a9,4A-a9′,B,C).

### 3.4. Attenuation of Oxidative Stress by RAPA in HFD-fed Group

#### 3.4.1. DHE Fluorescence

**Figure 5:**
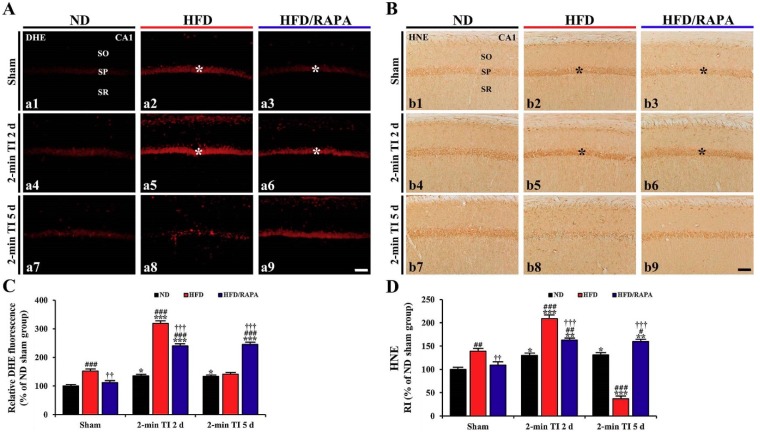
(**A**,**B**) DHE fluorescence staining (**A**) and HNE immunohistochemistry (**B**) in the CA1s of the ND-fed group (left columns), HFD-fed group (middle columns), and HFD/RAPA-fed group (right columns) at sham (a1–a3, b1–b3), 2 days (a4–a6, b4–b6), and 5 days (a7–a9, b7–b9) after 2-min TI. In the HFD/RAPA sham group, DHE fluorescence (a3) and HNE immunoreactivity (b3) in CA1 pyramidal cells (asterisks) were significantly deceased compared to the HFD/Vehicle sham group. In the HFD/RAPA 2-min TI groups at 2 days post-ischemia, DHE fluorescence (a6) and HNE immunoreactivity (b6) were significantly lower than the HFD 2-min TI group. Note that DHE fluorescence (a8) and HNE immunoreactivity (b8) in the HFD 2-min TI group at 5 days post-ischemia were very low due to death of CA1 pyramidal cells. Scale bar = 60 μm. (**C**,**D**) Quantitative analyses of DHE fluorescence (**C**) and HNE immunoreactivity (**D**) in CA1 pyramidal cells (*n* = 7/group). Relative ratios were calibrated as percentages, with the ND sham group designated as 100%. The bars indicate the means ± SEMs. * *p* < 0.05, ** *p* < 0.01; *** *p* < 0.001 cresyl violet each sham group, ^#^ *p* < 0.05, ^##^ *p* < 0.01, ^###^ *p* < 0.001 versus ND-fed group, ^†^ *p* < 0.05, ^††^ *p* < 0.01, ^†††^ *p* < 0.001 versus HFD-fed group.

In the ND sham group, the superoxide anion levels in CA1 pyramidal cells detected by DHE fluorescence were very low (Figure 5A-a1). In the ND 2-min TI group, the superoxide anion level was significantly increased (about 136%, *p* < 0.05, and 133%, *p* < 0.05 of the ND sham group, respectively) at 2 and 5 days post-ischemia compared to that in the ND sham group (Figure 5A-a4,5A-a7,C).

In the HFD sham group, the superoxide anion level in CA1 pyramidal cells was significantly higher (about 153% of the ND-sham group, *p* < 0.001) than that in the ND sham group (Figure 5A-a2,C). In the HFD 2-min TI group, superoxide the anion level was significantly increased (about 210% of the HFD sham group, *p* < 0.001) at 2 days post-ischemia (Figure 5A-a5,C) and significantly decreased at 5 days post-ischemia by about 93% of the HFD sham group, because CA1 pyramidal cells were damaged by 2 min of TI (Figure 5A-a8,C). 

In the HFD/RAPA sham group, a significant decrease (about 74% of the HFD sham group, *p* < 0.01) of superoxide anion level was observed in CA1 pyramidal cells (Figure 5A-a3,C). In the HFD/RAPA 2-min TI group, the superoxide anion level at 2 days post-ischemia was significantly lower (about 75% of the HFD 2-min TI group, *p* < 0.001) than that in the HFD 2-min TI group, and the decreased level sustained until 5 days post-ischemia (Figure 5A-a6,5A-a9,C).

#### 3.4.2. HNE Immunoreactivity

In the ND sham group, week HNE immunoreactivity was observed in CA1 pyramidal cells (Figure 5B-b1). In the ND 2-min TI group, HNE immunoreactivity was significantly increased (about 130%, *p* < 0.05, and 132%, *p* < 0.05 of the ND sham group, respectively) at 2 and 5 days post-ischemia compared to that in the ND sham group (Figure 5B-b4,4B-b7,D). 

In the HFD sham group, HNE immunoreactivity in the CA1 pyramidal cells was strong (about 139% of the ND sham group, *p* < 0.01) (Figure 5B-b2,D). In the HFD 2-min TI group, HNE immunoreactivity in the CA1 pyramidal cells was more increased (about 150% of the HFD sham group, *p* < 0.001) at 2 days post-ischemia compared to that in the HFD sham group (Figure 5B-b5,D). At 5 days post-ischemia, HNE immunoreactivity was significantly decreased by about 30% of the HFD sham group (*p* < 0.001) due to death of the CA1 pyramidal cells following 2-min TI (Figure 5B-b8,D).

In the HFD/RAPA sham group, HNE immunoreactivity in the CA1 pyramidal cells was significantly lower (about 78% of the HFD sham group, *p* < 0.01) than that in the HFD sham group (Figure 5B-b3,D). In the HFD/RAPA 2-min TI group, HNE immunoreactivity was significantly decreased (about 77% of the HFD 2-min TI group, *p* < 0.001) at 2 days post-ischemia, and the decreased HNE immunoreactivity was maintained until 5 days post-ischemia (Figure 5B-b6,5B-b9,D). 

### 3.5. Reduction of Neuroinflammation by RAPA in HFD-fed Group

#### 3.5.1. Iba-1^+^ Microglia

**Figure 6:**
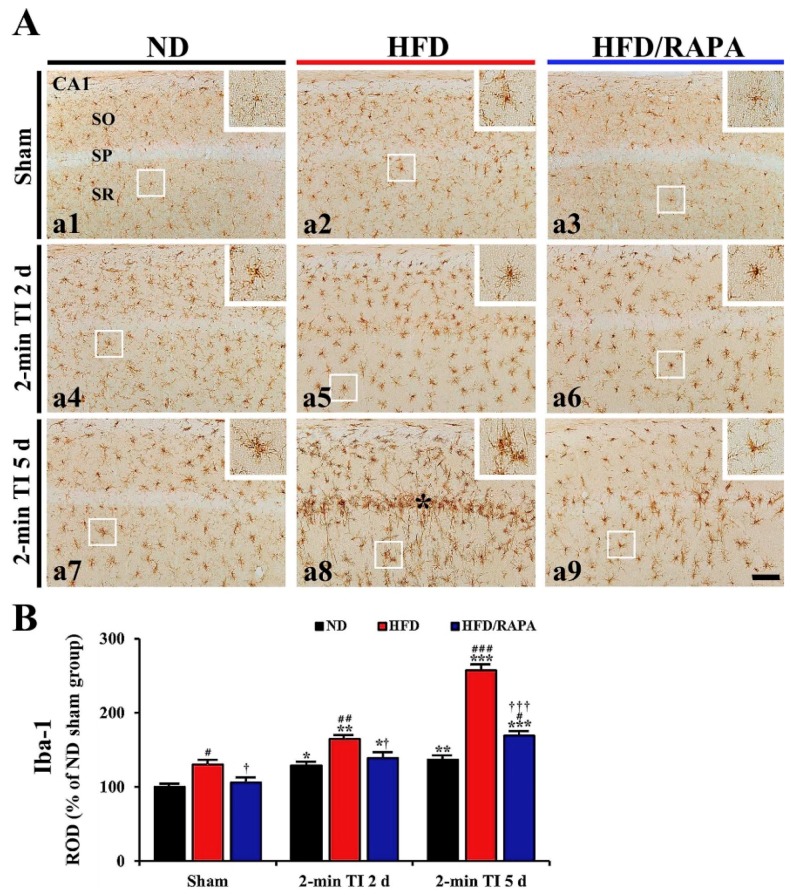
(**A**) Iba-1 immunohistochemistry in the CA1s of the ND-fed group (left column), HFD-fed group (middle column), and HFD/RAPA-fed group (right column) at sham (a1–a3), 2 days (a4–a6), and 5 days (a7–a9) after 2-min TI. In the HFD-fed group, Iba-1^+^ microglia were activated after 2-min TI, and the activated Iba-1^+^ microglia were aggregated in the stratum pyramidale (SP) (8, asterisk) at 5 days post-ischemia. However, the activation and aggregation of Iba-1^+^ microglia were markedly reduced in the HFD/RAPA-fed group after 2-min of TI. High magnification images are shown in white boxes. Scale bar = 60 μm. (**B**) Quantitative analysis of Iba-1^+^ structure in the CA1 (*n* = 7/group). ROD was calibrated as a percentage, with the ND sham group designated as 100%. The bars indicate the means ± SEMs. * *p* < 0.05, ** *p* < 0.01, *** *p* < 0.001 versus each sham group, ^#^ *p* < 0.05, ^##^ *p* < 0.01, ^###^ *p* < 0.001 versus ND-fed group, ^†^ *p* < 0.05, ^†††^ *p* < 0.001 versus HFD-fed group.

In the ND sham group, Iba-1^+^ microglia were mainly distributed in strata oriens and radiatum of the cells, and they had small amounts of cytoplasm with long branched processes (Figure 6A-a1). In the ND 2-min TI group, Iba-1^+^ microglia showed larger cell bodies with stouter processes, and the ROD of Iba-1^+^ structures was significantly increased (about 130%, *p* < 0.05, and 138%, *p* < 0.01 of the ND sham group, respectively) at 2 and 5 days post-ischemia (Figure 6A-a4,6A-a7,B). 

In the HFD sham group, Iba-1^+^ microglia displayed hypertrophic morphology compared to those in the ND sham group (Figure 6A-a2), and the ROD of Iba-1^+^ structures was significantly increased by about 31% of the ND sham group (*p* < 0.05) (Figure 6B). In the HFD 2-min TI group, Iba-1^+^ microglia were more hypertrophied after ischemia (Figure 6A-a5,6A-a8): at 5 days post-ischemia, strongly immunostained and hypertrophied Iba-1^+^ microglia were concentrated in the stratum pyramidale where the death of the CA1 pyramidal cells was discovered, and the ROD of Iba-1^+^ structures was very high (about 198% of the HFD sham group, *p* < 0.001) (Figure 6A-a8,B). 

In the HFD/RAPA sham group, the distribution pattern, morphology and ROD of Iba-1^+^ microglia were similar to the ND sham group (Figure 6A-a3,B). In the HFD/RAPA 2-min TI group, the ROD of Iba-1^+^ structures at 2 and 5 days post-ischemia was significantly low (about 81%, *p* < 0.05 and 66%, *p* < 0.001 of the HFD 2-min TI group, respectively) compared to the HFD 2-min TI group (Figure 6A-a6,6A-a9,B).

#### 3.5.2. IL-1β and TNF-α Immunoreactivity

**Figure 7:**
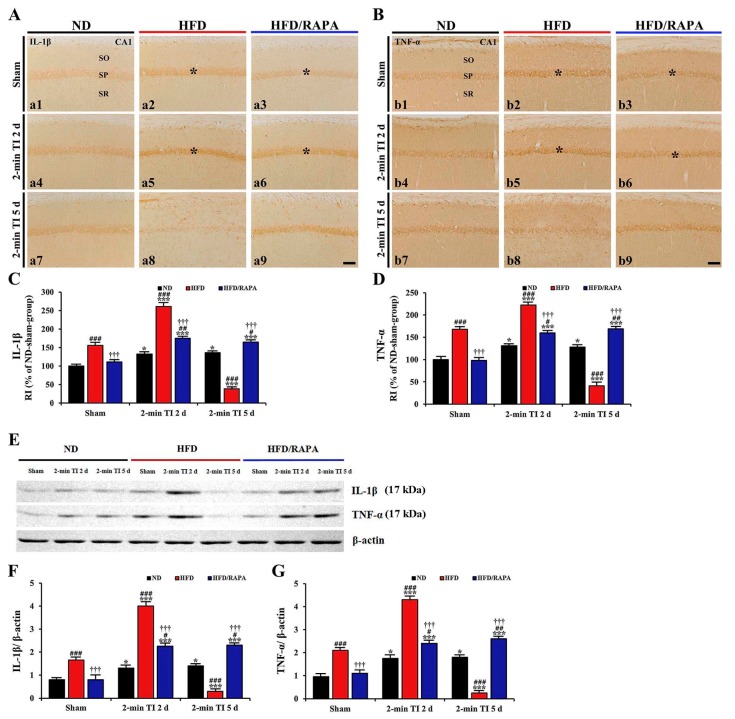
(**A**,**B**) Immunohistochemistry for IL-1β (**A**) and TNF-α (**B**) in the CA1s of the ND-fed group (left columns), HFD-fed group (middle columns), and HFD/RAPA-fed group (right columns) at sham (a1–a3, b1–b3), 2 days (a4–a6, b4–b6), and 5 days (a7–a9, b7–b9) after 2-min TI. IL-1β and TNF-α immunoreactivity in the HFD-fed group was significantly increased in CA1 pyramidal cells (asterisks) at sham (a2, b2) and 2 days (a5, b5) post-ischemia. Note that IL-1β and TNF-α immunoreactivity (asterisks) in the HFD/RAPA-fed group (a3, a6, b3, and b6) was significantly lower compared to the HFD/Vehicle-fed group. Scale bar = 60 μm. (**C**,**D**) Quantitative analyses of IL-1β (**C**) and TNF-α (**D**) immunoreactivity in CA1 pyramidal cells (*n* = 7/group). Relative immunoreactivity (RI) was calibrated as a percentage, with the ND sham group designated as 100%. The bars indicate the means ± SEM. ^*^p < 0.05, ^***^p < 0.001 versus each sham group, *^#^ p* < 0.05, *^##^ p* < 0.01, *^###^ p* < 0.001 versus ND-fed group, and *^†††^ p* < 0.001 versus HFD-fed group. (**E**) Western blotting for IL-1β (mature form, 17 kDa) and TNF-α (soluble form, 17 kDa) in the CA1s of the ND-fed, HFD-fed, and HFD/RAPA-fed groups at sham, 2 days, and 5 days after 2-min TI. (**F**,**G**) Semi-quantification of band intensities of IL-1β (**F**) and TNF-α (**G**) in the CA1 (*n* = 7/group). The bars indicate the means ± SEMs. * *p* < 0.05, *** *p* < 0.001 versus each sham group, ^#^ *p* < 0.05, ^##^ *p* < 0.01, ^###^ *p* < 0.001 versus ND-fed group, and ^†††^ *p* < 0.001 versus HFD-fed group.

In the ND sham group, weak IL-1β and TNF-α immunoreactivity was found in CA1 pyramidal cells (Figure 7A-a1,7B-b1). In the ND 2-min TI group, IL-1β and TNF-α immunoreactivity in CA1 pyramidal cells was significantly increased (about 133%, *p* < 0.05, and 131%, *p* < 0.05 of the ND sham group, respectively) at 2 days post-ischemia (Figure 7A-a4,7B-b4,C,D), and the increased IL-1β and TNF-α immunoreactivity was not changed until 5 days post-ischemia (Figure 7A-a7,7B-b7,C,D). 

In the HFD sham group, IL-1β and TNF-α, immunoreactivity in CA1 pyramidal cells was significantly higher (about 157%, *p* < 0.001, and 168%, *p* < 0.001, respectively) than that in the ND sham group (Figure 7A-a2,7B-b2,C,D). In the HFD 2-min TI group, IL-1β and TNF-α immunoreactivity was further significantly increased (about 167%, *p* < 0.001, and 133%, *p* < 0.001 of the HFD sham group, respectively) at 2 days post-ischemia (Figure 7A-a5,7B-b5,C,D), and each immunoreactivity was rarely detected in CA1 pyramidal cells at 5 days post-ischemia due to death of CA1 pyramidal cells following 2-min TI (Figure 7A-a8,7B-b8,C,D). 

In the HFD/RAPA sham group, IL-1β and TNF-α immunoreactivity in CA1 pyramidal cells was significantly decreased (about 71%, *p* < 0.001, and 58%, *p* < 0.001, respectively) compared to the HFD sham group (Figure 7A-a3,7B-b3,C,D). In addition, in the HFD/RAPA 2-min TI group, IL-1β and TNF-α immunoreactivity was significantly lower (about 63%, *p* < 0.001 and 71%, *p* < 0.001, respectively) than the HFD 2-min TI group at 2 days post-ischemia, and the immunoreactivity sustained until 5 days post-ischemia (Figure 7A-a6,7A-a9,7B-b6,7B-b9,C,D). 

#### 3.5.3. IL-1β and TNF-α Protein Levels

As shown in Figure 7E-G, IL-1β, and TNF-α protein levels were significantly altered in the CA1 after 2 min of TI. In the ND 2-min TI group, the IL-1β level was significantly increased (about 1.6-fold, *p* < 0.05, and 1.7-fold, *p* < 0.05 of the ND sham group, respectively) at 2 and 5 days post-ischemia. In addition, TNF-α level was significantly increased (about 1.7-fold, *p* < 0.05 and 1.8-fold, *p* < 0.05 of the ND sham group, respectively) at 2 and 5 days post-ischemia.

In the HFD sham group, IL-1β and TNF-α levels were about 2.1-fold (*p* < 0.001) and 2.2-fold (*p* < 0.001), respectively, of the ND sham group. In the HFD 2-min TI group, IL-1β and TNF-α levels were further significantly increased (about 2.4-fold, *p* < 0.001, and 2-fold, *p* < 0.001 of the HFD sham group, respectively) at 2 days post-ischemia and more significantly decreased (about 5.5-fold, *p* < 0.001, and 8.4-fold, *p* < 0.001 of the HFD sham group, respectively) at 5 days post-ischemia. 

In the HFD/RAPA sham group, the levels of IL-1β and TNF-α were significantly low (about 2.1-fold, *p* < 0.001, and 1.9-fold, *p* < 0.001 of the HFD sham group, respectively) compared to the HFD sham group. In the HFD/RAPA 2-min TI group, IL-1β and TNF-α levels were significantly decreased (about 1.7-fold, *p* < 0.001, and 1.8-fold, *p* < 0.001 of the HFD 2-min TI group, respectively) at 2 days post-ischemia, and the decreased levels were not altered until 5 days post-ischemia.

### 3.6. Inhibition of Abnormal mTOR Activation by RAPA in HFD-fed Group

#### 3.6.1. mTOR and p-mTOR Immunoreactivity

**Figure 8:**
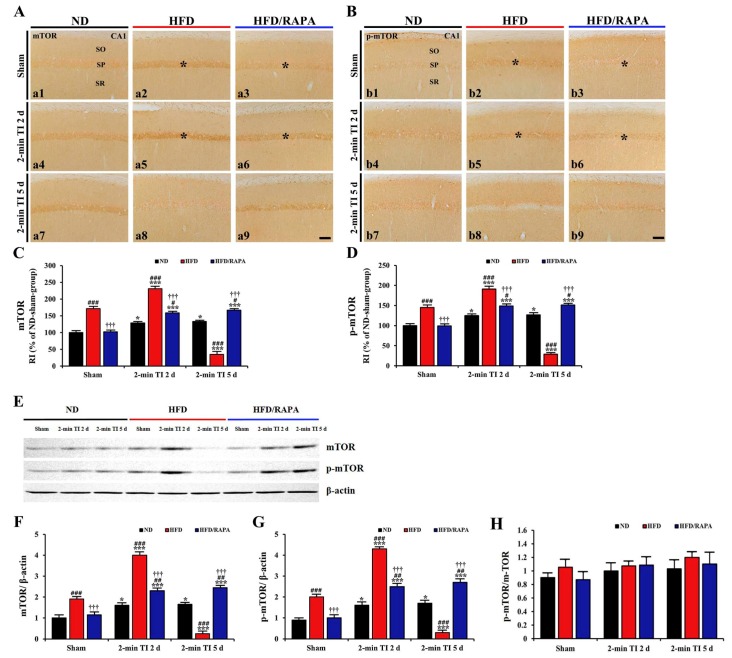
(**A**,**B**) Immunohistochemistry for mTOR (A) and p-mTOR (B) in the CA1s of the ND-fed (left columns), HFD-fed (middle columns), and HFD/RAPA-fed (right columns) groups at sham (a1–a3, b1–b3), 2 days (a4–a6, b4–b6), and 5 days (a7–a9, b7–b9) after 2-min TI. mTOR and p-mTOR immunoreactivity in the HFD-fed group was significantly higher in CA1 pyramidal cells (asterisks) than the ND-fed group at sham (a2, b2) and 2 days (a5, b5) after 2-min TI. In the HFD/RAPA-fed group, mTOR and p-mTOR immunoreactivity (asterisks) was significantly lower (a3, a6, b3, b6) compared to the HFD-fed group. Scale bar = 60 μm. (**C**,**D**) Quantitative analyses of mTOR (**C**) and p-mTOR (**D**) immunoreactivity in the CA1 pyramidal cells (*n* = 7/group). RI was calibrated as a percentage, with the ND sham group designated as 100%. The bars indicate the means ± SEMs. * *p* < 0.05, *** *p* < 0.001 versus each sham group, ^#^ *p* < 0.05, ^##^ *p* < 0.01, ^###^ *p* < 0.001 versus ND-fed group, and ^†††^ *p* < 0.001 versus HFD-fed group. (**E**) Western blotting of mTOR and p-mTOR in the CA1 of the ND-fed, HFD-fed, and HFD/RAPA-fed groups at sham, 2 days, and 5 days after 2-min TI. (**F**, **G**) Semi-quantification of band intensities of mTOR (**F**), p-mTOR (**G**), and p-mTOR/mTOR (**H**) ratios in the CA1s (*n* = 7/group). The bars indicate the means ± SEMs. * *p* < 0.05, *** *p* < 0.001 versus each sham group, ^##^ *p* < 0.01, ^###^ *p* < 0.001 versus ND-fed group, and ^†††^ *p* < 0.001 versus HFD-fed group.

In the ND sham group, weak mTOR and p-mTOR immunoreactivity was observed in CA1 pyramidal cells (Figure 8A-a1,8B-b1). In the ND 2-min TI group, mTOR and p-mTOR immunoreactivity was significantly increased (about 129%, *p* < 0.05, and 125%, *p* < 0.05 of the ND sham group, respectively) at 2 days post-ischemia, and the increased immunoreactivity sustained until 5 days post-ischemia (Figure 8A-a4,8A-a7,8B-b4,8B-b7,C,D). 

In the HFD sham group, mTOR and p-mTOR immunoreactivity in CA1 pyramidal cells was significantly higher (about 171%, *p* < 0.001, and 146%, *p* < 0.001 of the ND sham group, respectively) than the ND sham group (Figure 8A-a2,8B-b2,C,D). In the HFD 2-min TI group, mTOR, and p-mTOR immunoreactivity was further significantly increased (about 135%, *p* < 0.001, and 132%, *p* < 0.001 of the HFD sham group, respectively) at 2 days post-ischemia (Figure 8A-a5,B-b5,C,D). At 5 days post-ischemia, mTOR and p-mTOR immunoreactivity was significantly decreased (about 22%, *p* < 0.001, and 19%, *p* < 0.001 of the HFD sham group, respectively) due to 2-min TI-induced death of CA1 pyramidal cells (Figure 8A-a8,8B-b8,C,D). 

In the HFD/RAPA sham group, mTOR and p-mTOR immunoreactivity in CA1 pyramidal cells was significantly low (about 60%, *p* < 0.001, and 68%, *p* < 0.001 of the HFD sham group, respectively) compared to the HFD sham group (Figure 8A-a3,8B-b3,C,D). In the HFD/RAPA 2-min TI group, mTOR and p-mTOR immunoreactivity at 2 days post-ischemia was significantly lower (about 69%, *p* < 0.001, and 77%, *p* < 0.001 of the HFD 2-min TI group, respectively) than the HFD 2-min TI group, and, their immunoreactivity was maintained until 5 days post-ischemia (Figure 8A-a6,8A-a9,8B-b6,8B-b9,C,D).

#### 3.6.2. mTOR and p-mTOR Protein Levels

As shown in Figure 8E–G, mTOR and p-mTOR protein levels were changed in the CA1 after 2 min of TI. In the ND 2-min TI group, mTOR and p-mTOR levels were significantly increased (about 1.6-fold, *p* < 0.05, and 1.7-fold, *p* < 0.05 of the ND sham group, respectively) at 2 days post-ischemia, and each level was maintained until 5 days post-ischemia.

In the HFD sham group, mTOR, and p-mTOR levels were significantly higher (about 1.9-fold, *p* < 0.001, and 2.0-fold, *p* < 0.001 of the ND sham group, respectively) than those in the ND sham group. In the HFD 2-min TI group, mTOR and p-mTOR levels were further significantly increased (about 2.1-fold, *p* < 0.001, and 2.2-fold, *p* < 0.001 of the HFD sham group, respectively) at 2 days post-ischemia, and each level was very low at 5 days post-ischemia, when CA1 pyramidal cells were damaged by 2 min of TI.

In the HFD/RAPA sham group, mTOR and p-mTOR levels were significantly lower (about 1.7-fold, *p* < 0.001, and 2.0-fold, *p* < 0.001 of the HFD sham group, respectively) than that in the HFD sham group. In the HFD/RAPA 2-min TI group, each level was also lower (about 1.7-fold, *p* < 0.001, and 1.6-fold, *p* < 0.001 of the HFD 2-min TI group, respectively, *p* < 0.001) at 2 days post-ischemia compared to the HFD 2-min TI group, and the decreased levels were not changed until 5 days post-ischemia.

As shown in Figure 8H, ND TI, and HFD and/or HFD/RAPA TI had no effect on the protein expression of p-mTOR:mTOR ratio (*p* > 0.05). 

## 4. Discussion

Obesity is a condition characterized by an excessive accumulation of body fat that can lead to metabolic abnormalities, such as hyperlipidemia, hypercholesterolemia, and hyperglycemia, all of which can affect the pathophysiology of ischemic insults [33,34]. In this study, we set up a HFD-induced obese gerbil model to investigate effects of a 2-min TI on the neuronal damage in the hippocampus of HFD-induced obese gerbils. It has been shown that prolonged feeding with HFD is a well-established method to induce obesity in gerbils [34,35]. In HFD-fed gerbils, levels of blood glucose, serum total cholesterol, and triglyceride were significant increased, along with body and epididymal fat weight increases. In addition, we found that levels of serum leptin secreted from white adipose tissues known to control feeding behavior and energy balance were significantly increased in HFD-fed gerbils. It has been reported that circulating levels of leptin are significantly increased in obese individuals, probably because of leptin resistance [36].

There is growing evidence that HFD-induced obesity and metabolic abnormalities are closely associated with increases in ischemic brain injury. For example, obesity induced by HFD increases infarct volumes in brains following transient or permanent focal cerebral ischemia in rats [19,37] and mice [38,39]. Furthermore, we have recently demonstrated that HFD-induced metabolic abnormalities can accelerate and exacerbate neuronal death in the hippocampus and septum of gerbils subjected to 5 min of TI [40,41]. Contrary to the deleterious effects of obesity and metabolic abnormalities on ischemic brain injuries, it has been proven that a brief transient ischemic event confers cerebral ischemic tolerance against a subsequently longer or severer ischemic insult under normal bodily conditions [8,9]. In this regard, we have recently reported that the majority of CA1 pyramidal neurons can survive when normal gerbils are subjected to a subsequent 5 min of TI at 1 day following 2-min TI, suggesting that 2 min of TI confers ischemic tolerance to CA1 pyramidal neurons [6,14,42]. In this study, we found that 2 min of TI did not induce death of CA1 pyramidal neurons in ND-fed gerbils, as in our previous studies. However, massive loss of CA1 pyramidal neurons was observed in HFD-fed gerbils at 5 days after 2 min of TI. Based on relevant research and our present result, a brief episode of TI that can induce ischemic tolerance to CA1 pyramidal neurons under normal conditions might be able to the provoke death of pyramidal neurons in the CA1 under obesity’s conditions. 

Oxidative stress and neuroinflammation have been considered two major mechanisms underlying ischemic brain injury [43]. TI can result in ischemic neuronal death through oxidative damage induced by the excessive generation of reactive oxygen species and a strong neuroinflammatory response characterized by microglial activation and the release of inflammatory mediators [44,45]. However, it has been reported that oxidative stress induced by administration of diethyldithiocarbamate (a superoxide dismutase inhibitor) or an enhanced level of IL-1β following a brief episode (2 min) of TI protects neurons from a subsequent severer or longer TI [46,47]. In our present experiment, 2 min of TI increased HNE (an end-product of lipid peroxidation) immunoreactivity and DHE (an oxidant production marker) fluorescence, indicators of oxidative stress in CA1 pyramidal neurons of ND-fed gerbils. In this group, similarly, 2 min of TI elicited mild microglia activation and the mild expression of proinflammatory cytokines IL-1β and TNF-α in CA1 pyramidal neurons. Based on previous studies and our present results, 2 min of TI displays mild oxidative stress and neuroinflammation, suggesting that 2-min TI may induce ischemic tolerance in normal conditions. These mechanisms can protect neurons from subsequent longer or severer TI.

It has been well demonstrated that obesity is accompanied by chronically high oxidative stress and a strong inflammatory response in the brain that can aggravate ischemic brain injury [48,49]. In this study, our results showed that levels of oxidative stress indicators (HNE and DHE) and proinflammatory cytokines (IL-1β and TNF-α), as well as microglia activation were significantly higher in the CA1 of the HFD sham group than those in the ND sham group. This is consistent with previous studies showing that HFD-induced obesity could lead to significant increases in levels of lipid peroxidation products (HNE and malondialdehyde [MDA]) and TNF-α, as well as activation of microglia in the hippocampus of mice and rats [50,51]. Results of the present study also revealed that these factors in HFD-fed gerbils were severely increased after 2 min of TI compared to those in ND-fed gerbils. Preclinical studies have shown that levels of MDA and proinflammatory cytokines (IL-1β, IL-6 and TNF-α) as well as microglial activation in the ischemic brain tissues of HFD-induced obese mice and rats are significantly increased after mild (30 min) and severe (2 h) occlusion of the middle cerebral artery, respectively, compared to those in non-obese mice and rats [20,52]. Furthermore, we have recently reported that HFD-induced obese gerbils show significant increases in levels of MDA and proinflammatory cytokines (IL-1β and TNF-α) in the hippocampus following 5 min of TI compared to those in non-obese gerbils [34]. Thus, oxidative stress and neuroinflammation might be increased in HFD-fed obese gerbils before 2 min of TI compared to ND-fed non-obese gerbils. Such increases of oxidative stress and neuroinflammation might strongly contribute to neuronal death/loss following 2 min of TI in HFD-fed obese gerbils.

In this study, we found that protein levels of mTOR and p-mTOR were significantly higher in the CA1s of HFD-fed obese gerbils in pre- and/or post-ischemic phases than those in non-obese gerbils. It has been reported that 10 min of TI can lead to a significant increase of mTOR activation in the rat hippocampus [53] and that the inhibition of the mTOR pathway can attenuate ischemic brain injury by inhibiting lipid peroxidation, microglial activation, and the generation of proinflammatory cytokines (IL-1β and TNF-α) in rat models of transient (90 and 120 min) middle cerebral artery occlusion and cardiac arrest-induced transient (6 min) global ischemia [54,55,56]. On the other hand, in our current study, there was no significant change in p-mTOR/mTOR ratio between the sham ND- or HFD-fed gerbils and 2-min TI in ND- or HFD-fed gerbils, because the expression levels of both mTOR and p-mTOR were significantly increased, and the change patterns of them were very similar. Similar to our results, previous studies show significant increases of both mTOR and p-mTOR protein expressions in the rat’s cerebral cortex following 10 mins of TI [53], and in the gerbil striatum following 5 mins of TI [30]. Based on previous studies and our knowledge, we hypothesized that abnormal increases of mTOR and p-mTOR levels in the brain of HFD-induced obese gerbils in pre- and/or post-ischemic phases might be closely associated with severe increase of oxidative stress and neuroinflammation, along with neuronal death following 2 min of TI. To test our hypothesis, we evaluated whether the inhibition of abnormal mTOR activation by administration of RAPA, which is a highly selective mTOR inhibitor and penetrates the blood-brain barrier [57,58], could attenuate the death of CA1 pyramidal neurons in HFD-induced obese gerbils following 2 min of TI by inhibiting severe oxidative stress and neuroinflammation. Our results proved that the inhibition of abnormal mTOR activation by RAPA administration to HFD-induced obese gerbils significantly reduced obesity-induced severe increases in levels of oxidative stress indicators and proinflammatory cytokines and microglial activation during pre- and/or post-ischemic phases and attenuated CA1 pyramidal neuronal death induced by 2 min of TI, without affecting blood glucose levels, serum lipid concentrations, or leptin levels as expected. Although effects of RAPA on ischemic brain injury in HFD-induced obese animals have not fully been addressed, some studies have recently shown that inhibition of mTOR activation by RAPA administration in glucose-induced acute hyperglycemic and streptozotocin-induced chronic diabetic rats can alleviate the exacerbation of neuronal death in the cerebral cortex and CA1 following 5 min of TI without affecting plasma glucose levels, suggesting that exacerbating effects of metabolic abnormalities might be associated with the severer activation of mTOR under ischemic conditions [29,59]. Based on previous studies and our findings, one of major mechanisms of pyramidal neuronal death in CA1s, following 2 min of TI, in HFD-induced obese gerbils, could be abnormal activation of mTOR in pre- and/or post-ischemic phases. 

In summary, the results of this study clearly showed that 2 min of TI, which can induce cerebral ischemic tolerance in ND-fed non-obese gerbils, could provoke death of CA1 pyramidal neurons in HFD-induced obese gerbils. This might be closely associated with abnormal increase of mTOR activation-mediated severe oxidative stress and neuroinflammation in the hippocampal CA1 in pre- and/or post-ischemic phases under obesity’s conditions. Taken together, our findings strongly suggest that a brief period of TI known to confer ischemic tolerance in normal brains could lead to neuronal damage/death in obese patients.

## Supplementary Materials

The following are available online at https://www.mdpi.com/2073-4409/8/10/1126/s1: Figure S1 Negative control staining without primary antibody.
