# Supplementary figures and images for: A 2-Min Transient Ischemia Confers Cerebral Ischemic Tolerance in Non-Obese Gerbils, but Results in Neuronal Death in Obese Gerbils by Increasing Abnormal mTOR Activation-Mediated Oxidative Stress and Neuroinflammation
Source: Cells. 2019 Sep 22;8(10):1126. doi: 10.3390/cells8101126 (PMC6830098; doi:10.3390/cells8101126)

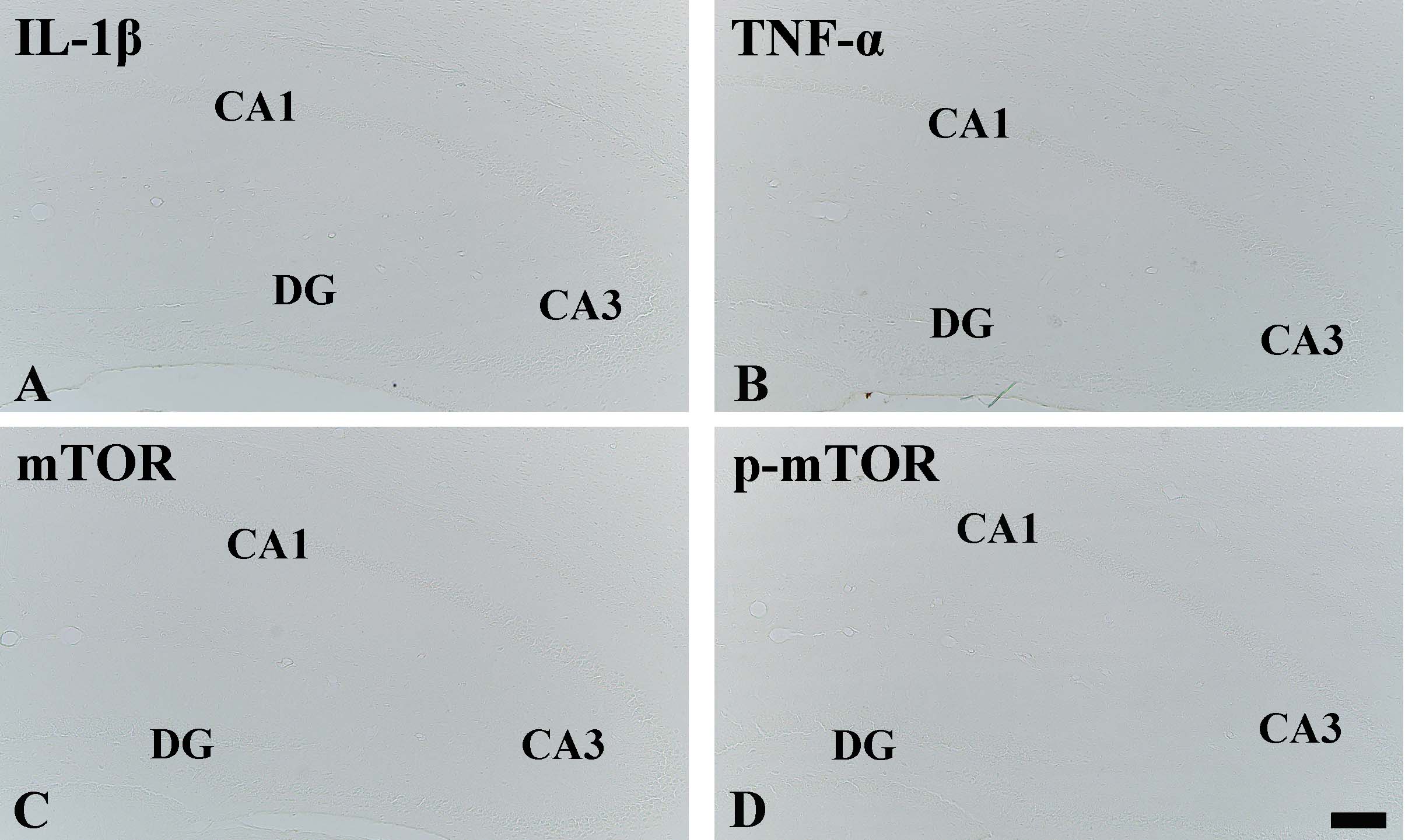

Supplement: Supplementary file 1 [file cells-08-01126-s001.zip › cells-590751-supplementary.jpg]
